# DMT Models the Near-Death Experience

**DOI:** 10.3389/fpsyg.2018.01424

**Published:** 2018-08-15

**Authors:** Christopher Timmermann, Leor Roseman, Luke Williams, David Erritzoe, Charlotte Martial, Héléna Cassol, Steven Laureys, David Nutt, Robin Carhart-Harris

**Affiliations:** ^1^Psychedelic Research Group, Centre for Psychiatry, Department of Medicine, Imperial College London, London, United Kingdom; ^2^The Computational, Cognitive & Clinical Neuroimaging Laboratory, Department of Medicine, Imperial College London, London, United Kingdom; ^3^GIGA-Consciousness and Neurology Department, Coma Science Group, University of Liège and University Hospital of Liège, Liège, Belgium

**Keywords:** DMT, *N*, *N*-Dimethyltryptamine, psychedelic, NDE, near-death experience, phenomenology, absorption, delusional ideation

## Abstract

Near-death experiences (NDEs) are complex subjective experiences, which have been previously associated with the psychedelic experience and more specifically with the experience induced by the potent serotonergic, *N*,*N*-Dimethyltryptamine (DMT). Potential similarities between both subjective states have been noted previously, including the subjective feeling of transcending one’s body and entering an alternative realm, perceiving and communicating with sentient ‘entities’ and themes related to death and dying. In this within-subjects placebo-controled study we aimed to test the similarities between the DMT state and NDEs, by administering DMT and placebo to 13 healthy participants, who then completed a validated and widely used measure of NDEs. Results revealed significant increases in phenomenological features associated with the NDE, following DMT administration compared to placebo. Also, we found significant relationships between the NDE scores and DMT-induced ego-dissolution and mystical-type experiences, as well as a significant association between NDE scores and baseline trait ‘absorption’ and delusional ideation measured at baseline. Furthermore, we found a significant overlap in nearly all of the NDE phenomenological features when comparing DMT-induced NDEs with a matched group of ‘actual’ NDE experiencers. These results reveal a striking similarity between these states that warrants further investigation.

“I’d be scared”.

“Scared of what?”

“Scared of dying, I guess. Of falling into the void”.

“They say you fly when you die”.

(Feature film: ‘Enter the Void’).

## Introduction

Near-death experiences (NDEs) are complex experiential episodes that occur in association with death or the perception that it is impending (Moody, 1975; Greyson, 1983). Prospective studies with cardiac arrest patients indicate that the incidence of NDEs vary between 2–18% depending on what criteria are used to determine them (Parnia et al., 2001; Van Lommel et al., 2001; Schwaninger et al., 2002; Greyson, 2003). Although there is no universally accepted definition of the NDE, common features include feelings of inner-peace, out-of-body experiences, traveling through a dark region or ‘void’ (commonly associated with a tunnel), visions of a bright light, entering into an unearthly ‘other realm’ and communicating with sentient ‘beings’ (Moody, 1975; Ring, 1980; Greyson, 1983; Martial et al., 2017). Reviewing the phenomenology of NDEs, we have been struck by similarities with the experience evoked by the classic serotonergic psychedelic *N*,*N*, Dimethyltryptamine (DMT) (Strassman et al., 1994; Strassman, 2001).

Commonly described features of the DMT experience include a feeling of transcending one’s body and entering into an alternative ‘realm’, an acoustic perception of a high pitched ‘whining/whirring’ sound during the onset of the experience, perceiving and communicating with ‘presences’ or ‘entities’, plus reflections on death, dying and the after-life (Sai-Halász et al., 1958; Strassman, 2001; Gouzoulis-Mayfrank et al., 2005). Furthermore, the reported vividness of both subjective experiences have led to NDE experiencers and DMT users describing the states they enter as ‘realer than real’ (for NDEs see Moody, 1975; Thonnard et al., 2013; for DMT see Strassman, 2001).

The term near-death exerpience (NDE) was coined by philosopher Raymond Moody more than 40 years ago (Moody, 1975). Remarkably, the overlap between the phenomenology of the classic serotonergic psychedelic experience and NDEs was highlighted by Moody himself more than 4 decades ago (Moody, 1975) and these similarities have formed the basis of a popular hypothesis on the pharmacology of NDEs, i.e., that endogenous DMT is released in significant concentrations during the dying process (Strassman, 2001), but see (Nichols, 2017) for a critique of this hypothesis. The psychological state produced by the DMT-containing Amazonian brew, ayahuasca (the literal translation of ‘ayahuasca’ from quechua is ‘the vine of the dead’ or ‘the vine of the soul’), has also been linked to themes of death and dying (Shanon, 2005) as have psychedelics in general (Millière, 2017), e.g., with the psychology of psychedelic-induced ‘ego-death’ being likened to that of actual death (Leary et al., 2008).

Both the psychedelic experience and NDEs appear to be sensitive to contextual factors such as prior psychological traits and state (‘set’), the environment (‘setting’) in which the experience unfolds (Metzner et al., 1965; Studerus et al., 2012) – plus the broader cultural context in which they are embedded (Wallace, 1959; Hartogsohn, 2017; Carhart-Harris et al., 2018). For example, controlled research has found that certain personality traits, e.g., ‘absorption’ and ‘neuroticism’ can predict the intensity and quality of a psychedelic experience (Studerus et al., 2012; Carhart-Harris et al., 2015, 2018; Carbonaro et al., 2016; Barrett et al., 2017) while readiness to ‘let go’ and quality of the environment also seems to be predictive of response (Carhart-Harris et al., 2018). In a similar fashion, the prevalence and nature of NDEs appear to be sensitive to environmental, demographic and personality variables, such as etiology and prognosis of the NDE, age, absorption and a propensity to report paranormal experiences (Kohr, 1983; Greyson, 2003). Cultural factors are presumed to influence the psychedelic experience (Carhart-Harris et al., 2018) and have been found to influence the content of NDEs, (Kellehear, 1993; Kellehear et al., 1994).

The near-death experience has been associated with long-term positive changes in psychological well-being and related outcomes; more specifically, greater concern for others, reductions in distress associated with the prospect of dying, increased appreciation for nature, reduced interest in social status and possessions, as well as increased self-worth have all been observed and/or described post NDEs (Noyes, 1980; Ring, 1980; Groth-Marnat and Summers, 1998). Relatedly, recent results from studies with psychedelic compounds have shown similar long-term positive changes. For example, reduced death anxiety (Grob et al., 2011; Gasser et al., 2015; Griffiths et al., 2016; Ross et al., 2016), pro-ecological behavior (Forstmann and Sagioglou, 2017; Nour et al., 2017) and nature relatedness (Lyons and Carhart-Harris, 2018), significant clinical improvements in depressed patients (Osório et al., 2015; Carhart-Harris et al., 2016; Palhano-Fontes et al., 2018) and recovering addicts (Johnson et al., 2014; Bogenschutz et al., 2015; Bogenschutz and Johnson, 2016) and lasting improvements in psychological well-being in healthy populations (Griffiths et al., 2011; Carhart-Harris et al., 2017) have all been observed. Thus, overlap between near-death and psychedelic experiences may extend beyond the acute experience into longer-term psychological changes.

While the subjective effects of DMT have been researched in the past (Strassman et al., 1994; Gouzoulis-Mayfrank et al., 2005), they have tended to be collapsed into broad categories or dimensions of experience (e.g., visual, somatic and emotional effects) as determined by standard ‘altered states of consciousness’ rating scales (Strassman et al., 1994; Studerus et al., 2010). The degree to which DMT specifically induces near-death type experiences has never been directly measured, however.

This current within-subjects, placebo-controlled study aimed to directly measure the extent to which intravenous DMT given to healthy volunteers in a laboratory setting could induce a near-dear type experience as determined by a standard NDE rating scale (Greyson, 1983). Importantly, we also aimed to address how these experiences compared with a sample of individuals who claim to have had ‘actual’ near-death experiences. To our knowledge, this is the first time that the relationship between DMT experiences and (non-drug-induced) NDEs has ever been formally addressed.

We hypothesized that DMT would induce near-death type experiences of an equivalent intensity to those seen previously in the context of ‘actual’ NDEs, and to a significantly greater extent than in the placebo condition. Based on aforementioned work on NDEs, we also hypothesized that age, personality and a propensity toward delusional thinking would correlate with DMT-induced near-death experiences.

## Materials and Methods

### Experimental Design

Thirteen healthy volunteers participants (6 female, 7 male, mean age: 34.4, *SD*: 9.1 years) participated in a fixed-order, placebo-controlled, single blind study, approved by the National Research Ethics Service (NRES) Committee London – Brent and the Health Research Authority (HRA). This study was carried out in accordance with the recommendations of Good Clinical Practice guidelines, Declaration of Helsinki ethical standards and the NHS Research Governance framework. All subjects gave written informed consent in accordance with the Declaration of Helsinki. The study was sponsored and approved by Imperial College London’s Joint Research and Compliance Office (JRCO) and the National Institute for Health Research/Wellcome Trust Imperial Clinical Research Facility gave site-specific approval for the study. The research was conducted under a Home Office license for research with Schedule 1 drugs. Study procedures consisted of screening and 2 dosing sessions, separated by 1 week.

### Screening

Participants were recruited via word-of-mouth and received an information sheet detailing all study procedures prior to the screening visits. Informed consent was obtained before screening, which consisted of routine physical tests (routine blood tests, electrocardiogram, blood pressure, heart rate, neurological examination) a psychiatric interview and examination. The main exclusion criteria were: an absence of experience with a classic psychedelic drug (e.g., LSD, psilocybin, DMT, ayahuasca), current or previously diagnosed psychiatric illness, immediate family history of psychotic disorder, excessive use of alcohol (>40 weekly units), blood or needle phobia and a significant medical condition rendering volunteers unsuitable for participation (e.g., diabetes, heart condition). Tests for drug abuse and pregnancy (when applicable) were performed on screening and study days and participants were required to abstain from using psychoactive drugs at least 7 days prior to study participation.

Following screening, participants were enrolled for 2 dosing sessions in which placebo and DMT were administered. Questionnaires were completed electronically prior to the dosing sessions – which served as baseline correlation measures. Following each dosing sessions, participants completed questionnaires enquiring about subjective experiences during the DMT and placebo sessions. The Greyson NDE scale (Greyson, 1983) served as the primary outcome measure.

### Study Procedures and Participants

Both dosing sessions took place at the National Institute of Health Research (NIHR) Imperial Clinical Research Facility (CRF). Participants rested in reclined position in a dimly lit room, while low volume music was played in the background in order to promote calm during the session (Johnson et al., 2008). Electroencephalogram (EEG) recordings took place before and following administration of DMT and placebo (the relevant findings concerning EEG results will be reported elsewhere).

Participants received one of four doses of DMT fumarate (three volunteers received 7 mg, four received 14 mg, one received 18 mg and five received 20 mg) via intravenous route in a 2 ml sterile solution over 30 s, followed by a 5 ml saline flush lasting 15 s. Placebo consisted of a 2 ml sterile saline solution, which followed the same procedure (Strassman and Qualls, 1994). During the first dosing session, all participants received placebo, and 1 week later, DMT. Participants were unaware of the order in which placebo and DMT were administered but the research team was (i.e., single-blind study design). The order was fixed in this way to promote safety by developing familiarity with the research team and environment prior to receiving DMT, and to avoid potential carry over effects from receiving DMT first (particularly as the experience is associated with lasting psychological effects – see section “Introduction”).

Participants reported feeling the subjective effects of DMT immediately after the 30 s injection or during the flush which came soon after it. Effects peaked at 2–3 min and gradually subsided, with only residual effects felt 20 min post administration. Volunteers were discharged to go home by a study psychiatrist at least 1 h after administration and once all study procedures were completed. Participants were asked to message a member of the research team in order to confirm their safe return and well-being. To ensure safety, each volunteer was supervised by two researchers and the study physician throughout the dosing session.

### Main Outcomes and Measures

#### Acute Outcomes

In order to determine the degree to which DMT induces near-death type experiences, the Near-Death Experience scale (NDE scale; Greyson, 1983) was completed retrospectively once the effects of DMT and placebo had subsided. This is the most widely used scale for NDEs; it was first constructed from a questionnaire based on a sample of 67 participants who had undergone 73 NDEs in total (Greyson, 1983). The NDE scale consists of 16 items, resulting in a total score representing the global intensity of the experience as well as scores for four subscales: (1) *Cognitive*, (2) *Affective*, (3) *Transcendental*, and (4) *Paranormal*. A total score higher or equal to 7 is considered the threshold for a NDE (Greyson, 1983).

The overlap between drug-induced NDEs and other relevant psychological phenomena associated with psychedelic drugs was also addressed. Two additional measures were included for this purpose, namely: The Ego Dissolution Inventory (EDI) (Nour et al., 2016) and the Mystical Experiences Questionnaire (MEQ) (Maclean et al., 2013). The EDI contains 8 items and a mean score on all 8 is calculated for a single EDI factor. The MEQ contains 30 items and yields a total score consisting of the average of all items as well as four subscales: *Mystical, Positive Mood, Transcendence of Time and Space* and *Ineffability* (Maclean et al., 2013; Barrett et al., 2015).

#### Additional Measures

##### Correlations with personality trait absorption, delusional thinking and age

Questionnaires completed at baseline (before study visits) were used to assess the relationship between personality, suggestibility, delusional thinking and age with the magnitude of the NDE scores. Previous research has identified that the personality trait absorption and reports of so-called ‘paranormal’ phenomena (e.g., telepathic communication, out-of-body experiences) are positively correlated with the NDE scores, while age is negatively correlated with NDE scores (Kohr, 1983; Greyson, 2003). Because reports of paranormal experiences have been associated with magical ideation and schizotypy (Brugger and Taylor, 2003) we used the Peters’ Delusion Inventory (PDI) (Peters et al., 2004) to establish the relationship between this construct and NDE scores. The PDI is a measure of delusional thinking in the general population and contains items related to paranormal phenomena (e.g., belief in telepathy, witchcraft, and voodoo) as well as strength of belief and level of distress associated with these (Peters et al., 2004).

Participants were also asked to complete the modified version of the Tellegen Aborption Questionnaire (MODTAS) (Tellegen and Atkinson, 1974). Pearson-product moment correlations were used to test for relationships between the relevant variables and main outcomes (i.e., the relationship between absorption, delusional thinking and age with NDE scores was analyzed). In order to adhere to statistical principles, one-tailed analyses were performed in cases in which there were clear, evidence-informed hypotheses about the direction of correlations, otherwise two-tailed tests were performed.

##### Comparison to ‘actual’ NDE group

In order to address the degree of overlap between our results and the features typically reported by people who have reported ‘actual’ NDEs, we conducted a separate comparison with gender and age matched sample of individuals who had completed the NDE scale (and scored above the established cutoff for an NDE) from a few months to 15 years (mean time = 7 years, *SD* = 6 years) after experiencing a life-threatening episode. This sample was defined as the NDE group (7 female, 6 male, mean age = 36.62, *SD* = 7.65). NDE experiencers were recruited via the Coma Science Group (GIGA-Consciousness, University and University Hospital of Liège, Belgium) and the International Associations for Near-Death Studies (IANDS France and Flanders). Participants were mailed a questionnaire that included items about socio-demographic (gender, age at interview) and clinical (time since NDE) characteristics. They were then asked to respond to the Greyson NDE scale (Greyson, 1983).

### Statistical Analysis

To compare the acute effects of DMT with those of placebo, repeated measures Analysis of Variance (ANOVA) were performed separately on data from the NDE scale and the MEQ, using condition (DMT vs. placebo) and questionnaire subscales as the factors of interest. *Post hoc* paired *t*-tests were then performed to compare DMT vs. placebo. In order to dissect the relationship between the DMT and near-death experiences, each item of the NDE scale was also subjected to paired *t-*tests (DMT vs. placebo). The comparison between the DMT state and ‘actual’ near-death experiences was made by conducting paired *t*-tests for each NDE scale item, as well as its subscales and total score.

Overlap between MEQ and NDE scale scores was assessed via Pearson-Product Moment Correlation on the main score of the difference between DMT and placebo for both scales, and the same procedure was performed between the EDI and the NDE scale. Independent correlation analyses were performed using the main score of the NDE (DMT-placebo) and each of the MEQ sub-factors in order to determine which of the MEQ sub-factors shows the strongest association with the total NDE scale scores.

Effect sizes were calculated using Cohen’s *d* for all paired *t-*tests. Separate Pearson-Product Moment Correlations were performed using each of the variables collected at baseline vs. the total NDE score. All analyses that involved less than 15 comparisons used Bonferroni-correction for multiple comparisons, while those equal/above 15 comparisons used False-Discovery Rate (FDR) correction. All *t*-tests were performed under two-tailed analyses.

## Results

### DMT Induces Near-Death Type Experiences

**FIGURE 1:**
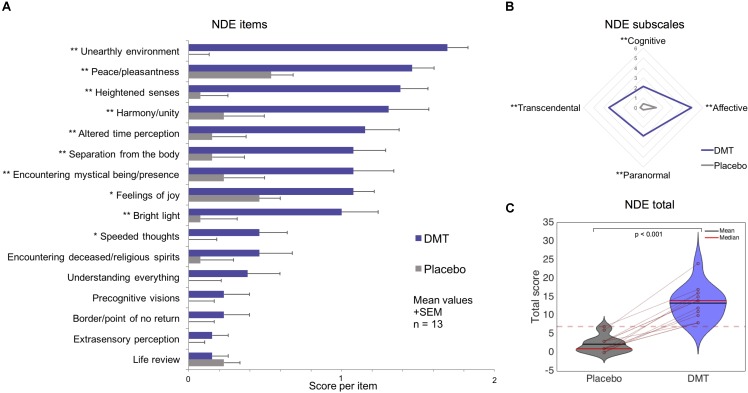
Near-Death Experience (NDE) scale results for DMT vs. placebo. Significantly larger scores for DMT versus placebo were found for **(A)** 10 out of 16 items of the NDE scale; **(B)** all NDE subscales; and **(C)** the NDE scale total (the dotted red line corresponds to the conventional threshold for determining an NDE) (^∗^*p* < 0.05, ^∗∗^*p* < 0.01, corrected for multiple comparisons).

All participants scored above the conventional cutoff (above or equal to 7) for a (DMT-induced) near-death (type) experience (Greyson, 1983). One of the 13 participants had a total score of 7 following placebo. The Analysis of Variance revealed a significant main effect of *condition* [*F*(1,12) = 118.95, *p* = 1.39e^-7^], a main effect of NDE *subscale* [*F*(4,48) = 59.36, *p* = 5.39e^-18^] and an interaction between *condition* and NDE *subscale* [*F*(3,36) = 11.92, *p* = 1.4e^-5^]. *Post hoc t*-tests revealed all NDE subscales and the total NDE score to be significantly increased under DMT compared to placebo (*p* < 0.01 Bonferroni corrected) and the comparison of the total score was significantly higher for DMT compared to placebo (*t* = 10.91, df = 12, *p* = 1.39e^-7^, Cohen’s *d* = 3.09). Paired *t*-tests on each of the 16 items comprising the NDE scale were performed in order to assess the specific phenomenological features of the DMT experience. Fifteen of the 16 items were scored higher under DMT than placebo and 10 of these reached statistical significance after correction (*p* < 0.01 FDR-corrected) (**Figure 1**). These results show that near-death experience phenomena were significantly enhanced following DMT administration.

### Ego-Dissolution Inventory (EDI) and Mystical Experience Questionnaire (MEQ)

A paired comparison using the EDI revealed highly significant larger total scores for DMT compared to placebo (*T* = 6.98, df = 12, *p* = 1.47e^-5^, Cohen’s *d* = 2.67). Analysis on the Mystical Experience Questionnaire (MEQ) (Maclean et al., 2013) revealed a main effect of *condition* [*F*(1,12) = 133.27, *p* = 7.43e^-8^], a main effect of MEQ sub-scale or *factor* [*F*(3,36) = 4.66, *p* = 0.007] and a *condition*^∗^*factor* interaction [*F*(3,36) = 3.35, *p* = 0.03]. *Post hoc t*-tests revealed that all MEQ factors were significantly higher following DMT compared to placebo (*p* < 0.001) and the total score was also significantly increased under DMT vs. placebo (*t* = 10.11, df = 12, *p* = 3.17e^-7^, Cohen’s *d* = 3.81).

**FIGURE 2:**
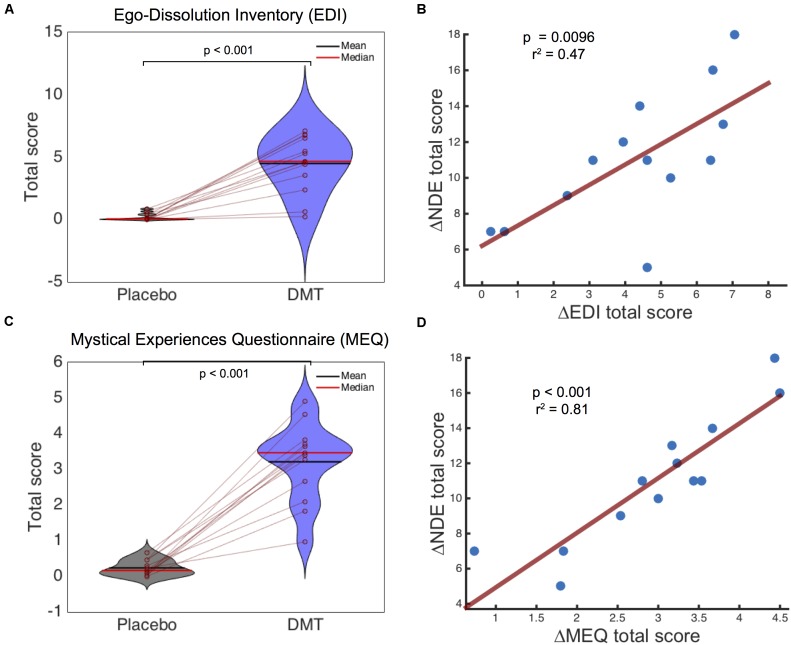
Ego-Dissolution Inventory (EDI) and Mystical Experiences Questionnaire (MEQ) scores and relationship with Near-Death Experience (NDE) scale scores. Significantly higher scores were found for DMT compared to placebo for **(A)** EDI and **(C)** MEQ scores. A highly significantly positive correlation was found between the NDE scale and **(B)** EDI and **(D)** MEQ scores following DMT administration.

The difference between DMT and placebo scores for the NDE scale, EDI and MEQ were used for correlation analysis. These analyses revealed a significant correlation for both the EDI (*r* = 0.69, *r*^2^ = 0.47, *p* = 0.0096) and MEQ (*r* = 0.90, *r*^2^ = 0.81, *p* = 2.60e^-5^) with the NDE scale (**Figure 2**). Independent Pearson Product-Moment correlation analyses were performed using each of the different MEQ factors separately against the NDE total score. A significant relationship was revealed between the *Mystical* (*r* = 0.9, *r*^2^ = 0.81, *p* < 0.001) and *Transcendence of Time and Space* (*r* = 0.72, *r*^2^ = 0.51, *p* = 0.006) factors and the NDE score, while *Positive Mood* (*r* = 0.67, *r*^2^ = 0.45, *p* = 0.013) and *Ineffability* (*r* = 0.63, *r*^2^ = 0.40, *p* = 0.021) did not survive multiple-comparison correction (0.05/4 = 0.0125). Overall these results indicate a high overlap between near-death type experiences, ego-dissolution and mystical-type experiences induced by DMT. With specific regards to the mystical experience, MEQ factors *Mystical* and *Transcendence of Time and Space* were most strongly associated with the DMT-induced near-death type experiences.

### Correlations With Personality, Delusional Ideation and Age

**FIGURE 3:**
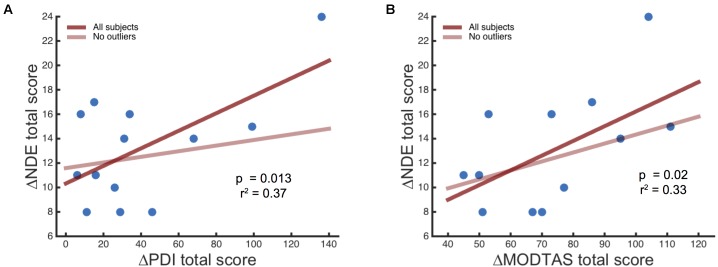
Association between the NDE scale scores following DMT administration and baseline measures of **(A)** delusional beliefs (PDI) and **(B)** Absorption scores (MODTAS). The transparent red line shows the slope of the *r*-value discarding the outlier.

The personality trait absorption has been associated with NDEs in patient populations (Twemlow and Gabbard, 1984), therefore a Pearson Product-Moment Correlation analysis was performed using the scores of Tellegen Absorption Scale (MODTAS) and NDE questionnaire. Alleged ‘paranormal’ experiences have also been associated with higher NDE scores (Kohr, 1983; Greyson, 2003) and delusional thinking (measured through the PDI) and younger age have also been shown to correlate with NDE scores. Here, correlation analysis revealed a positive relationship between baseline PDI scores and NDE scores after DMT (*r* = 0.61, *r*^2^ = 0.37, *p* = 0.013, one-tailed). Similarly, baseline MODTAS absorption scores showed the same relationship (*r* = 0.57, *r*^2^ = 0.33, *p* = 0.02, one-tailed); however, this did not survive multiple comparison correction (0.05/4 = 0.0125). Performing the same analysis after excluding an outlier revealed a trend relationship between NDE scale and MODTAS scores (*r* = 0.45, *r*^2^ = 0.21, *p* = 0.07, one-tailed) but no significant relationship between the PDI and the NDE scale (*r* = 0.19, *r*^2^ = 0.04, *p* = 0.28, one-tailed) (**Figure 3**), nor between age and NDE (*r* = -0.36, *r*^2^ = 0.13, *p* = 0.16).

### Comparison to ‘Actual’ Near-Death Experiences

**FIGURE 4:**
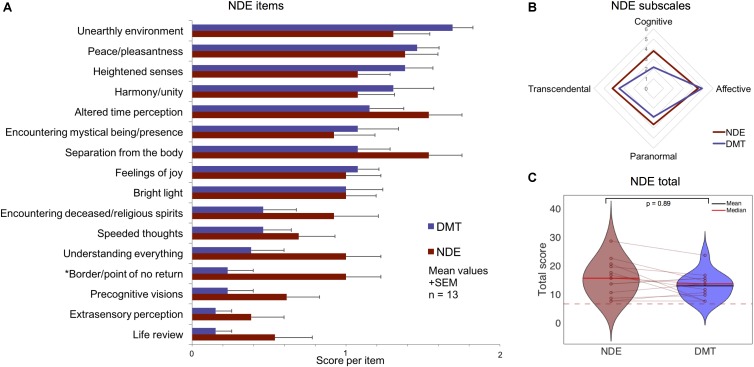
Comparison of near-death experience (NDE) features in ‘actual’ NDEs and the DMT experience using a standard NDE scale (Greyson, 1983). No significant differences were found between DMT administration and ‘actual’ NDEs for **(A)** 15 out of 16 NDE scale items; **(B)** all NDE subscales; and **(C)** the NDE scale total score (The dotted red line corresponds to the threshold for an NDE). (^∗^*p* < 0.05 uncorrected).

A separate analysis was performed using a gender/age matched sample of volunteers who reported having gone through an ‘actual’ NDE. Participants were selected based on scores above a standard cut-off on the NDE scale of 7 points (Greyson, 1983). **Table 1** and **Figure 4** displays the results from separate *t*-tests performed on each item, subscales and total score. Overall the results show that the total NDE scores induced by DMT are comparable to those given by the ‘actual’ NDE group (*t* = 1.85, df = 12, *p* = 0.089, Cohen’s *d* = 0.49).

**Table 1:** Comparison of NDE scale features between ‘actual’ NDEs and following DMT administration (matched samples) (^∗^*p* < 0.05 uncorrected).

	NDE scale mean scores (*SD*)		Effect size (Cohen’s *d*)
Items	NDE	DMT	NDE vs. DMT
**Cognitive subscale**	3.77 (2.24)	2.15 (1.77)	0.80
*Did time seem to speed up or slow down?*	1.54 (0.78)	1.15 (0.80)	0.49
*Were your thoughts speeded up?*	0.69 (0.85)	0.46 (0.66)	0.30
*Did scenes from your past come back to you?*	0.54 (0.88)	0.15 (0.38)	0.57
*Did you suddenly seem to understand everything?*	1.00 (0.82)	0.38 (0.77)	0.78
**Affective subscale**	4.46 (2.57)	4.85 (1.63)	-0.18)
*Did you have a feeling of peace or pleasantness?*	1.38 (0.77)	1.46 (0.52)	-0.12
*Did you have a feeling of joy?*	1.00 (0.82)	1.08 (0.49)	-0.11
*Did you feel a sense of harmony or unity with the universe?*	1.08 (0.76)	1.31 (0.95)	0.00
*Did you see, or feel surrounded by, a brilliant light?*	1.00 (0.71)	1.00 (0.91)	0.00
**‘Paranormal’ subscale**	3.62 (1.89)	2.85 (1.52)	0.36
*Were your senses more vivid than usual?*	1.08 (0.76)	1.38 (0.65)	-0.44
*Did you seem to be aware of things going on elsewhere, as if by extrasensory perception (ESP)?*	0.38 (0.77)	0.15 (0.38)	0.38
*Did scenes from the future come to you?*	0.62 (0.77)	0.23 (0.60)	0.56
*Did you feel separated from your body?*	1.54 (0.78)	1.08 (0.76)	0.60
**Transcendental subscale**	4.15 (2.30)	3.46 (1.66)	0.34
*Did you seem to enter some other, unearthly world?*	1.31 (0.85)	1.69 (0.48)	-0.55
*Did you seem to encounter a mystical being or presence, or hear an unidentifiable voice?*	0.92 (0.95)	1.08 (0.95)	-0.17
*Did you see deceased or religious spirits?*	0.92 (1.04)	0.46 (0.78)	0.50
*Did you come to a border or point of no return?*	1.00 (0.82)	0.23 (0.60)	1.07^*^
**Total**	16 (6.28)	13.31 (4.55)	0.49


All of the subscales ratings were also comparable between NDE and DMT conditions. The only feature showing a significant difference was the item “*Did you come to a border or point of no return”* – which was scored higher by the NDE group compared to the DMT condition (*t* = 2.74, df = 12, *p* = 0.018 – uncorrected, Cohen’s *d* = 1.07); however, this result did not survive correction for multiple comparisons (see **Table 1** for differences on specific items of the NDE scale). Overall, comparable NDE scores can be seen for the ‘actual’ NDE group and the DMT condition.

## Discussion

This study sought to examine the degree to which features commonly reported in NDEs are elicited by the potent serotonergic psychedelic DMT in a placebo-controlled study. Results revealed that *all* 13 participants scored above the standard threshold for an NDE in relation to their DMT experiences (Greyson, 1983) and 15 of the 16 NDE items were rated significantly higher under DMT compared to placebo, with 10 of these reaching statistical significance after multiple testing correction.

Especially strong overlap was seen between DMT-induced near-death type experiences and mystical-type experiences (MEQ, Griffiths et al., 2006, 2016; Kometer et al., 2015), with the *Mystical* factor of the MEQ (which contains items such as “sense of being at a spiritual height” and “experience of oneness or unity with objects and/or persons in your surroundings”) showing the highest relationship with NDE total scores. Intriguingly, DMT-induced NDE scores were significantly correlated with baseline-measured delusional thinking. Perhaps most interesting of all however, when these DMT data were compared with those from a matched sample of ‘actual’ NDEs, a comparable profile was evident, with few discernable differences between the experiences of the actual NDE cases and those induced by DMT. Taken together, these results reveal a striking similarity between the phenomenology of NDEs and experiences induced by the classic serotonergic psychedelic, DMT.

As reported above, 10 of 16 NDE items were scored significantly higher under DMT than placebo. Items that did not survive multiple comparison correction (experiences of extrasensory perception, life-review, precognition of future events, increased speed of thoughts and seeing deceased people/relatives) are also items that are less commonly endorsed in ‘actual’ NDEs (Schwaninger et al., 2002; Greyson, 2003; Vanhaudenhuyse et al., 2009). The *Affective* subscale of the NDE scale was scored particularly highly under DMT, and emotion is also a prominent feature of actual NDEs (Greyson, 1983, 2003; Schwaninger et al., 2002; Martial et al., 2017). Subtle differences that were apparent between the DMT condition and NDEs (e.g., the experience of entering an unearthly realm was enhanced for DMT vs. actual NDEs, whereas coming to a point of no return was scored higher in actual NDEs compared with the DMT experience) may be explainable by the very different contexts in which these experiences occur (e.g., DMT was given here with prior screening, psychological preparation and consent in a safe laboratory setting vs. an NDE occuring during an illness or unexpected accident) as much as differences due to the inducers themselves or their associated neurobiologies.

It is important to acknowledge that the phenomenology of NDEs is still a matter of some investigation. Initially, 15 NDE features were proposed when the term was first coined – based on interviews with more than 50 NDE ‘experiencers’ (Moody, 1975); after this, 5 core features were identified via structured interviews (Ring, 1980), and subsequently Greyson (1983) identified 16 features using statistical methods in a study comprising 73 different NDEs.

It has recently been shown that the temporal sequence of events unfolding during an NDE is highly variable between people and no prototypical sequence was identifiable in a sample of 154 participants, although four main dimensions were relatively consistent, namely: ‘out-of-body experiences,’ ‘seeing a bright light,’ ‘encountering spirits/people,’ and a ‘feeling of peace’ (Martial et al., 2017). The potential heterogeneity of NDEs cautions us to consider how intra and inter-individual variables, cultural characteristics and the environmental and psychological context in which they take place may influence the content of experiences as well as whether and how they are reported.

Personality has previously been associated with response to psychedelics (Studerus et al., 2012; Hartogsohn, 2016; Barrett et al., 2017). For example, trait ‘absorption’ has been shown to be predictive of sensitivity to psychedelics (Studerus et al., 2012), and the same has also been shown in relation to the intensity of ‘actual’ NDEs (Twemlow and Gabbard, 1984). Absorption has been linked to a serotonin 2A receptor polymorphism associated with greater signaling (Ott et al., 2005); thus, it is intriguing to consider whether abnormal serotonergic functioning may contribute to both psychedelic and NDEs (Carhart-Harris and Nutt, 2017). Here, we saw a trend toward absorption predicting DMT-induced NDE but this relationship did not quite reach statistical significance, perhaps due to insufficient statistical power or that the use of different doses might have masked this effect.

It has been previously shown that the reported intensity of NDEs is associated with a tendency to report so-called ‘paranormal’ experiences (Kohr, 1983; Greyson, 2003). Our results show that baseline delusional thinking (as measured by Peters’ Delusion Inventory), was strongly associated with NDE scores. One possible interpretation of this is that – like people with paranormal beliefs – people with higher than average delusional thinking are more emphatic in their endorsement of NDE phenomena as they view it as less at odds with their pre-existing belief systems and perhaps even see it as ‘evidence’ for metaphysical and/or mystical beliefs which they already endorse. Recent findings (Martial et al., 2018) have found a strong relationship between ‘fantasy proneness’ and NDE phenomena reported by individuals in situations in which there has been no genuine threat to their lives (e.g., in meditative states or under intense psychological distress). These results support the view that individuals’ traits and beliefs might strongly influence the appearance of such phenomena in a range of different contexts, which could account for our current findings.

Relatedly, we found a strong relationship between scores of DMT-induced near-death type and mystical-type experiences. More specifically, we found a strong association between the total NDE score and the MEQ factors ‘*Mystical*’ and ‘*Transcendence of Time and Space.’* The Mystical factor corresponds to items pertaining to an experience of unity or continuity between self/ego and the external world (known elsewhere as ‘dissolved ego-boundaries’- Nour et al., 2016), an intuitive feeling of so-called ‘sacredness’, and the experience of gaining insights into ‘ultimate truths’. The factor *Transcendence of Time and Space* corresponds to experiences of loss of one’s usual spatial and temporal orientation and a sense of vastness, continuity and eternity (Pahnke and Richards, 1970; Maclean et al., 2013). The strong overlap between these facets of mystical-type and NDEs may be due to similar items featuring in both scales (e.g., items pertaining changes in time perception, experiences of unity, feelings of peace) which could be seen as evidence of their strong phenomenological overlap – but there are also some items that are distinct between the scales, e.g., feelings of being separated from one’s one body, encountering beings or presences.

Recent work has consistently shown that the occurrence of mystical-type experiences is predictive of long-term therapeutic benefit from psychedelics (Maclean et al., 2011; Garcia-Romeu et al., 2014; Griffiths et al., 2016; Carhart-Harris et al., 2017, 2018; Roseman et al., 2018) and similar mechanisms may be at play in relation to improved mental well-being post NDE (Moody, 1975; Noyes, 1980; Ring, 1980; Groth-Marnat and Summers, 1998). It is pertinent to ask therefore, what common features shared between these states may be responsible for mediating the apparent long-term psychological benefits that follow them. Evidence suggests that that the experience of unity – which some have claimed is an inevitable counterpart to ego-dissolution (Nour et al., 2016) – may be the core component binding them both. The so-called ‘unitive experience’ was originally identified as the core component of the mystical experience by its most influential scholar, Walter Stace (Stace, 1960) and it is also recognized in descriptions of the ‘peak experience’ – an overtly secular equivalent of the so-called ‘mystical experience’ introduced by Maslow (1959), as well as the ‘oceanic feeling’ coined by Romain Rolland in conversation with Sigmund Freud, who believed the feeling to be regressive, recapitulating the state of consciousness inhabited by infants prior to the development of the ego (Freud, 1920). It is possible that complete ego-dissolution and the parallel unitive experience that accompanies it may be the common factor that can bridge between these different states and is also responsible for the longer-term psychological benefits associated with them. Another recent thought, is that a return of the brain to ‘criticality’ (Atasoy et al., 2017), albeit temporarily, may offer a reminder of one’s closeness with nature (Carhart-Harris, 2018) and so what is left afterwards is as much an epistemic ‘reminder’ as anything else.

Detailed interviewing techniques could serve to improve our characterization of the phenomenology of both the DMT and NDE states (Petitmengin, 2006; Petitmengin and Lachaux, 2013), and future studies of the psychedelic state could benefit from adopting a more dynamic sampling approach, i.e., by attempting to detail the temporal unfolding of the experience – as has been done recently in the context of NDEs (Martial et al., 2017) and elsewhere in relation to stream of consciousness (Smallwood and Schooler, 2015). We predict that improved ‘capture’ of certain transient experiences within a broader psychedelic experience may help finesse our understanding of its psychology and associated neurobiology.

Rudimentary neurobiological models of the NDE have existed for almost 30 years, and have tended to lay emphasis on abnormal serotonergic and medial temporal lobe activity (Morse et al., 1989; Saavedra-Aguilar and Gómez-Jeria, 1989; Britton and Bootzin, 2004) – consistent with the predominantly serotonergic pharmacology of classic psychedelics (Nichols, 2016) as well as findings from fMRI (Tagliazucchi et al., 2014; Carhart-harris et al., 2016) and depth EEG recordings of human brain activity under psychedelics which implicate the medial temporal lobes (Carhart-Harris, 2007; Carhart-Harris and Nutt, 2014). Given strong associations between the temporal lobes and more specifically, medial temporal lobe structures, and unusual psychological experiences such as those featuring within NDEs (Carhart-Harris, 2007), we predict that the medial temporal regions may be implicated in some of the content and emotion-rich epochs that arise within the psychedelic state, such as complex imagery, entity encounters, and vivid autobiographical recollections. The relinquishment of top-down cortical control over temporal lobe activity may be an important component of this mechanism (Alonso et al., 2015; Kaelen et al., 2016; Timmermann et al., 2017).

### Limitations

It is important to acknowledge this study’s limitations. The dose of DMT was not uniform for all participants due to this study being part of a dose-finding pre-study ahead of a larger EEG-fMRI study with DMT. The lower doses of DMT may have actually depreciated the true strength of the similarities between the DMT state and the near-death experience therefore, and we should also acknowledge that individuals included within the ‘actual’ NDE sample had to give NDE-scale scores above a specific threshold, whereas this pre-requisite wasn’t stipulated for our DMT sample.

Another limitation is that we cannot discount the influence of order effects as placebo sessions were always performed first (to avoid carryover and promote comfort for the DMT session) and thus, exposure to the NDE scale post placebo may have primed participants to experience NDE-like phenomena ahead of their DMT sessions – although this seems unlikely given the volume of other measures and time between sessions. The positive scores on the NDE scale might also reflect a general tendency to endorse ‘anything unusual’ in relation to the psychedelic experience, particularly as psychedelics have been shown to promote suggestibility (Carhart-Harris et al., 2015). Contradicting this explanation, however: (1) participants’ mental state had largely returned to baseline by the time they completed the NDE scale, (2) responses to the different dimensions of the NDE scale resembled those seen in relation to actual NDEs, and (3) an open interview performed post experience (but before questionnaires were completed) promoted careful reflection on the details of the DMT state. That one participant scored on the threshold for an NDE in the placebo session suggests that the NDE scale may have a somewhat liberal threshold for determining NDEs – and thus may warrant revision.

As both psychedelics and the NDE phenomena appear to be strongly influenced by contextual factors (Kellehear et al., 1994; Carhart-Harris et al., 2018), it could be argued that significant differences may exist regarding phenomenological features between both experiences. Nonetheless, considering the strong overlap on the items of the NDE scale, the study of such differences might require the use of other methods addressing nuances not explored here (e.g., the use of questionnaires addressing contextual factors and qualitative studies).

We should also consider that although the study of the phenomenology of NDEs and psychedelic experiences may inform on each other in a reciprocal way, using one psychological phenomenon to model another, particularly if they are as abstract as the near-death and psychedelic experiences are, these may be fraught with problems (see Langlitz, 2017 for a detailed reflection on psychedelics as models of other mental and psychiatric phenomena). For this reason, better understanding their (presumed) shared neurobiology may provide the necessary bedrock to ground the science of these fascinating states.

## Conclusion

This study aimed to examine potential overlap between the phenomenology of NDEs and those associated with the potent serotonergic psychedelic DMT. Results revealed an intriguingly strong overlap between specific and broad features of these states, with DMT participants scoring high on a standard measure of NDEs and in a comparable way to people reporting bona fide NDEs, with only subtle differences that might relate more to obvious contextual differences than anything to do with the specific inducers themselves.

Indeed, these present results suggest that certain contextual factors (e.g., delusional thinking and personality trait absorption) can significantly mediate both the intensity and quality of the DMT-induced NDE-like experiences, advancing the notion that – as with the psychedelic experience more broadly (Carhart-Harris et al., 2018) – the intensity and content of NDEs are context-dependent. This study’s findings warrant further investigation to address the putatively strong overlap between the phenomenology and neurobiology of DMT (and other psychedelic) experiences and ‘actual’ near-death experiences, particular given some of the scientifically problematic yet influential claims that have been made about NDEs (Alexander, 2012).

Better understanding of both the psychology and neurobiology of dying (Borjigin et al., 2013) – e.g., by using psychedelics to model it – may have implications for how we view this most inevitable and universal phenomenon, potentially promoting a greater familiarity with and healthy acceptance of it.

‘*By meditating on death, we paradoxically become conscious of life. How extraordinary it is to be here at all. Awareness of death can jolt us awake to the sensuality of existence*.’ (Batchelor, 1998).

## Author Contributions

CT designed and conducted the research, analyzed the data, and wrote the paper. LR and LW conducted the research. DE overlooked medical and health standards for the research. CM, HC, and SL contributed the data from the NDE group. DN sanctioned the research. RC-H designed the research and wrote the paper.

## Conflict of Interest Statement

The authors declare that the research was conducted in the absence of any commercial or financial relationships that could be construed as a potential conflict of interest.
